# Microsurgical resection of previously embolized recurrent cerebellopontine angle AVM

**DOI:** 10.3171/2020.10.FOCVID2057

**Published:** 2021-01-01

**Authors:** Ehsan Dowlati, Kelsi Chesney, Vikram V. Nayar

**Affiliations:** Department of Neurosurgery, MedStar Georgetown University Hospital, Washington, DC

**Keywords:** microsurgical resection, arteriovenous malformation, embolization, recurrence, Onyx

## Abstract

This is the case of a ruptured Spetzler-Martin grade II arteriovenous malformation (AVM) located in the cerebellopontine angle and draining into the transverse sinus. The AVM was initially treated with staged embolization using Onyx (ev3 Neurovascular). However, recurrence was noted and treatment with microsurgical resection was undertaken. The authors present technical nuances of the approach and strategies for microsurgical resection of a previously embolized recurrent AVM with the aid of intraoperative indocyanine green angiography. Follow-up after endovascular treatment is critical, and curative treatment with microsurgical resection can be achieved with low morbidity in such AVMs as demonstrated by this case.

The video can be found here: https://youtu.be/LMpz_YTFC0g

## Transcript

**0:21 Introduction.** This is a case of a previously ruptured recurrent cerebellopontine angle AVM that was initially treated with endovascular embolization.

**0:29 Patient History and Exam.** The patient is a 66-year-old male who presented to an outside institution after sudden headache, right-sided numbness, and dizziness.

On exam, he awakened to voice and was oriented times three. His pupils were equal and reactive. He had a right abducens palsy and right facial droop. He had mild paresis in the right upper and lower extremities and numbness to light touch on the right side.

**0:52 Preoperative Imaging.** Patient underwent a CT head that showed a 5 × 3.5–cm right cerebellar hemorrhage with extension into the ventricles causing obstructive hydrocephalus. CTA head showed a suspected AVM with a draining vein toward the right transverse-sigmoid junction.

**1:09 Surgical Treatment.** In the setting of symptomatic posterior fossa hemorrhage, patient underwent emergent external ventricular drain placement and suboccipital decompression with hematoma evacuation.

**1:19 Diagnostic Angiogram.** Subsequently, the patient underwent a diagnostic cerebral angiogram shown here to better characterize the AVM. This demonstrated a Spetzler-Martin grade II AVM due to eloquent location. The nidus measured 18 × 18 mm, fed by the superior cerebellar artery and anterior inferior cerebellar artery on the right. It contained flow-related perinidal aneurysms, which are known to be associated more with posterior circulation AVMs.^1,2^ It drained through a dilated cerebellar vein into the right transverse sinus. Patient underwent endovascular treatment at the originating institution.

**1:57 Embolization Stage 1.** Stage 1 of endovascular treatment occurred with embolization of two branches of the superior cerebellar artery feeding the AVM. This occluded about 80% of the AVM with 1.6 ml of Onyx-34 (ev3 Neurovascular).

**2:12 Embolization Stage 2.** Stage 2 took place the following day with embolization of the anterior inferior cerebellar artery branches and associated perinidal aneurysm. Notably on postembolization injections, embolic material did not penetrate the AVM nidus completely, making recurrence a possibility.^3^

**2:33 Postembolization Course.** Patient recovered from his hemorrhage and was discharged to rehab. His neurological exam was significant for dysarthria, stable right abducens palsy, right facial droop, and left hemiparesis as a complication of nontarget embolization of a lateral pontine artery.

Patient was referred to our institution afterwards and seen for a follow-up angiogram at 4½ months.

**2:58 Follow-Up Angiogram and Imaging.** Follow-up angiogram showed that most of the AVM nidus remained occluded. However, as shown in these oblique views of right vertebral artery injection, recurrence of arteriovenous shunting was present with supply from the right superior cerebellar artery draining into the right transverse sinus. Notably, there was now also some supply through the right tentorial artery making this a pial AVM with additional dural supply.

A preoperative MRI was obtained with T2 axial sequences shown here noting the encephalomalacia from prior hemorrhage and the nidus with Onyx-casted arteries. A CTA obtained showed these embolized vessels; however, recurrent feeding arteries were not well appreciated given the artifact.

Given the recurrence, treatment options were discussed, including observation, repeat embolization, radiosurgery, or microsurgical resection. The decision was made to proceed with microsurgical resection for its ability to provide the most definitive and immediate treatment for this low-grade previously ruptured AVM.

**4:02 Retrosigmoid Approach.** We chose a retrosigmoid approach to access the CP angle AVM. Patient was positioned three-quarter prone with the head affixed to a rigid 3-point Mayfield head holder. The head is rotated away from the side of the lesion and the neck is flexed. A lazy S–shaped incision is marked out behind the ear away from his prior midline incision and 2 cm behind the pinna. The incision is made through the subcutaneous tissue and subperiosteal dissection of the soft tissue and muscle is undertaken to expose the subocciput and asterion.

A retrosigmoid craniotomy is performed at the transverse-sigmoid junction and the dura is opened in a cruciate fashion as shown. This allows exposure to the CP angle fissure for release of CSF, which allows optimal visualization lateral to the brainstem under the tentorium.

After the craniotomy is done and connected with the prior craniectomy defect, the microscope is brought in for improved visualization and illumination. The dural opening is completed and the dural leaflets are tacked up with 4-0 Nurolon sutures.

**5:05 Exposure of the CPA.** The right CPA fissure is followed and opened sharply, freeing arachnoid from the tentorium, as well as thickened adhesions formed from the prior hemorrhage. This allows for the cerebellum to relax away from the tentorium.

Starting inferiorly, the cerebellomedullary fissure is opened, separating the arachnoid from the right posterior inferior cerebellar artery and lower cranial nerves. A combination of microforceps and microscissors are used to lyse the adhesions. Dissection is moved superiorly toward the trigeminal nerve. Feeding vessels infiltrated with Onyx become visible, and they are followed toward the nidus. The superior cerebellar artery is identified superior deep to the nidus along with an embolized branch.

**5:53 Resection of AVM.** The AVM nidus is seen in the right CP angle posterior to the trigeminal root exit zone. Onyx embolic material was present throughout much of the nidus as well as in various feeding vessels seen here. The upper aspect of the AVM nidus remained patent and arterialized. The arterialized draining vein entering the tentorium and draining into the right transverse sinus is identified. Intraoperative indocyanine green angiography is performed to visualize the AVM and its draining vein.

**6:19** Using gentle spreading of the microforceps and bimanual technique, feeding arteries of the AVM are identified and dissected. Using bipolar cautery and sharp dissection, a plane is cleared around the AVM nidus and away from the cerebellar parenchyma. We coagulate and divide small arterial feeders to the AVM.

**6:41** Predominantly, these feeders were from branches of the superior cerebellar artery. Feeding arteries containing Onyx were also coagulated and sharply divided at a point adjacent to the nidus to allow release from surrounding structures. Here, an embolized perinidal aneurysm is seen as we dissect adjacent to the trigeminal nerve.

**7:03** Starting superficial to deep, feeding arteries both contributing to the nidus and previously embolized are disconnected in systematic fashion, while preserving other vessels and critical structures of the CP angle. After circumferential dissection around the AVM nidus, the draining vein remains arterialized. We inspect around the nidus, noting that embolized arteries may be left behind as they are no longer feeding the AVM. Another indocyanine green intraoperative video angiogram is performed and compared with the one just prior to resection.

**7:41** The remaining arterial supply to the arterialized vein now originated within the tentorium. A clip is placed and the infratentorial portion of the AVM is removed and sent as specimen to pathology. Next, the tentorium surrounding the vein is coagulated and divided. After this, the vein appears bluish in color and is no longer arterialized. A final fluorescence video angiogram confirms no residual nidus with no arterial blood flow in the vein present. A 7-mm straight Yaşargil clip is then placed across the draining vein at the tentorium.

**8:16** Closure. Hemostasis is achieved and copious irrigation is applied to the resection bed. The dura is closed in a watertight fashion with a synthetic dural patch graft. The bone flap was reaffixed with titanium plates and screws, and the muscles and scalp were closed in a standard multilayered fashion.

**8:34 Postoperative Imaging.** Immediate postoperative angiogram showed complete resolution of the AVM, as shown in this AP and lateral injection of the right vertebral artery.

Postoperative CT head showed expected postsurgical changes.

**8:49 Postoperative Course.** Postoperatively, the patient remained neurologically stable and discharged home on POD 5. He recovered well and remained without new symptoms at 3-month and 6-month follow-up. Final pathology confirmed an arteriovenous malformation.

**9:04 Conclusion.** In summary, follow-up is important in endovascularly treated AVMs,^4^ particularly those of the brainstem and CP angle.^5,6^ Microsurgical resection of previously embolized recurrent AVMs is safe and effective and is the optimal treatment of choice after recurrence of low-grade AVMs in the posterior fossa, providing definitive treatment.

## Author Contributions

Primary surgeon: Nayar. Assistant surgeon: Dowlati. Editing and drafting the video and abstract: Dowlati, Chesney. Critically revising the work: all authors. Reviewed submitted version of the work: all authors. Approved the final version of the work on behalf of all authors: Dowlati. Supervision: Nayar.
